# Pneumomédiastin spontané: un diagnostic rare et bénin du jeune adulte (à propos d’un cas)

**DOI:** 10.11604/pamj.2021.38.238.27011

**Published:** 2021-03-05

**Authors:** Fatima Zahra Yousfi, Sofia Guerrouj, Afafe Thouil, Hatim Kouismi

**Affiliations:** 1 Centre Hospitalier Universitaire Mohammed VI Oujda, Faculté de Médecine et de Pharmacie, Oujda, Maroc

**Keywords:** Pneumomédiastin spontanée, syndrome de Boerhaave, emphysème sous-cutanée, à propos d’un cas, Spontaneous pneumomediastinum, Boerhaave´s syndrome, subcutaneous emphysema, case report

## Abstract

Le pneumomédiastin spontané se définit par la présence d´air au niveau du médiastin survenant en dehors de tout contexte traumatique, iatrogène ou d´une maladie pulmonaire sous-jacente. C´est une affection rare, qui survient chez le jeune adulte et l´adolescent. Son principal risque est d´être confondu avec un tableau de fistule aéro-digestive, conduisant parfois à la réalisation d´investigations et de mesures thérapeutiques inutiles, voire délétères. Nous rapportons le cas d´un pneumomedisatin spontané survenu chez une femme jeune au décours d´un effort de vomissement.

## Abstract (en)

Spontaneous pneumomediastinum is defined as the presence of air in the mediastinum. It is not iatrogenic, due to a trauma or underlying lung disease. It is a rare condition affecting young adults and adolescents. The main risk of diagnostic error is due to the fact that features can be confused with those of aerodigestive fistula, sometimes leading to investigations and unnecessary and sometimes deleterious therapeutic measures. We here report a case of spontaneous, vomiting-induced pneumomediastinum in a young woman.

## Introduction

Le pneumomédiastin spontané, ou emphysème médiastinal, est une affection peu fréquente, rarement diagnostiquée avant 18 ans, affectant surtout l´adulte jeune (30 ans), de sexe masculin [1]. Il représente un cas sur 7 000 à 12 000 admissions à l´hôpital [2]. Il survient en dehors de tout contexte traumatique, ou iatrogène, et en dehors de toute pathologie pulmonaire sous-jacente [2].

## Patient et observation

Il s´agit d´une patiente de 22 ans, sans antécédents pathologiques notables, qui présente depuis 04 mois avant son admission à l´hôpital des vomissements alimentaires récurrents non rythmés par les repas, associés à une douleur retro-sternale à type de brulure et d´ une dyspnée stade III de la mMRC (Modified Medical Research Council) sans autres signes respiratoires ou extra-respiratoires associés, évoluant dans un contexte d´altération de l´état général et d´amaigrissement chiffré à 14kg durant 04 mois. Examen clinique trouvait une patiente consciente, en bon état général OMS à 1, dyspnéique stable sur le plan hémodynamique et respiratoire. Emphysème sous cutanée cervico-thoracique, avec crépitations neigeuses caractéristiques à la palpation du cou.

**Figure 1:**
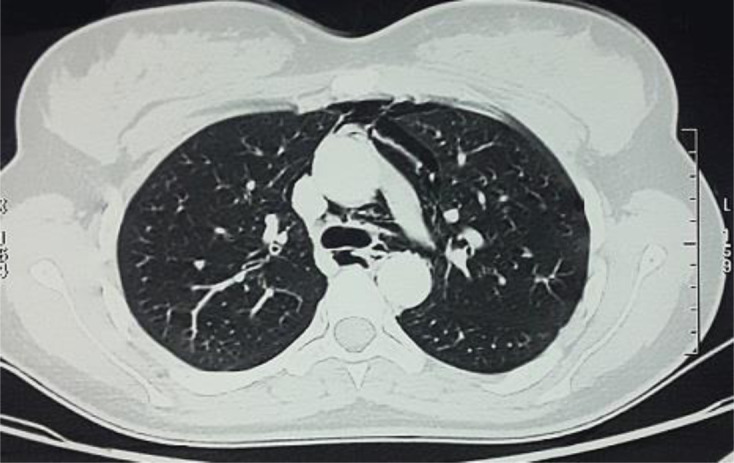
TDM thoracique en fenêtre parenchymateuse, coupe axiale: pneumomédiastin de grande abondance au niveau des parois thoraciques antérieure latérales et postérieure gauche

Un bilan radiologique était réalisé fait d´une radio-thoracique qui était normale, alors que le scanner thoracique objectivait un pneumomédiastin de grande abondance associée à un emphysème des parties molles cervicale et de l´hemichamps thoracique gauche, avec absence d´extravasation de produit de contraste après opacification (Figure 1). Devant ce tableau clinico-radiologique une endoscopie digestive était faite aux urgences afin d´écarter une rupture œsophagienne ainsi qu´une fibroscopie bronchique souple à la recherche d´une fistule oeso-tracheal.

**Figure 2:**
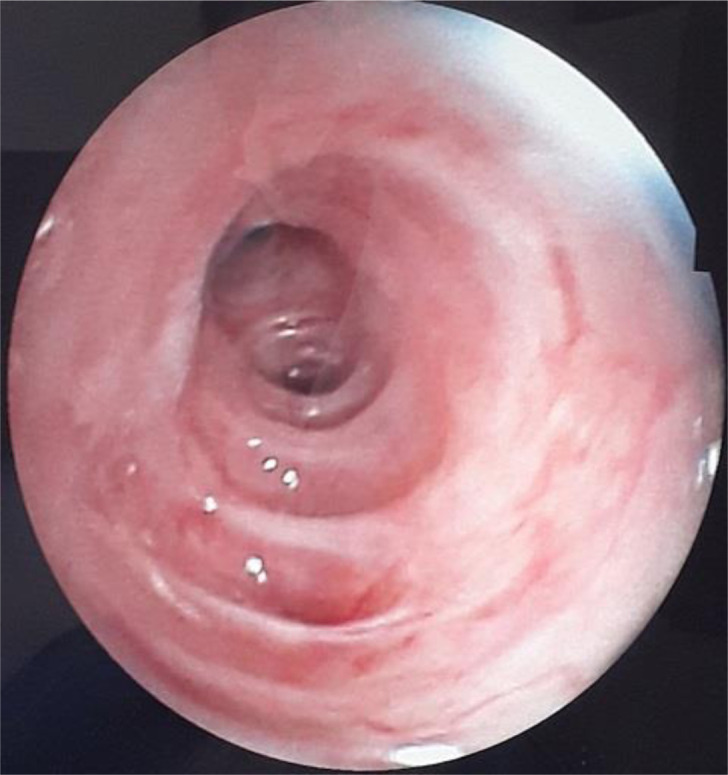
fibroscopie bronchique souple: aspect endoscopique normale de la trachée avec absence de fistule œoso-tracheale

**Figure 3:**
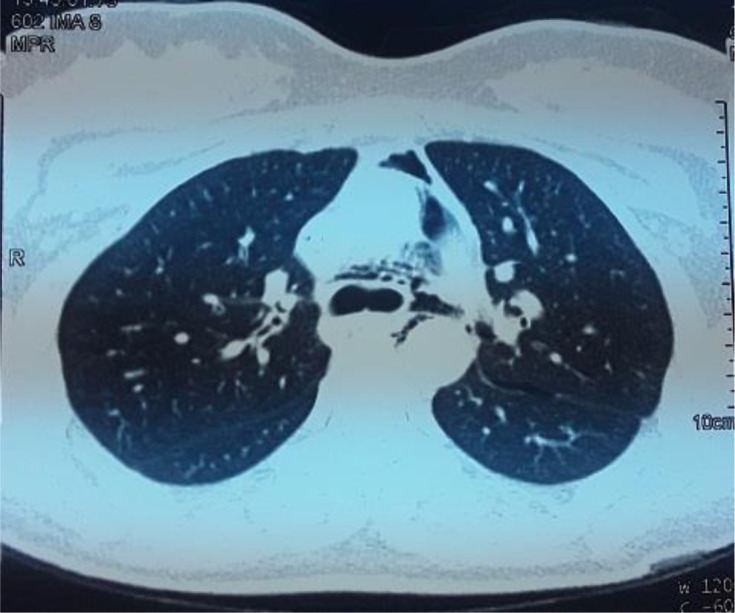
TDM thoracique en fenêtre parenchymateuse, coupe axiale: évolution radiologique du pneumomédiatsin marquée par la diminution de l´épanchement aérique médiastinal

La fibroscopie bronchique souple était sans particularité n´ayant pas objectivé de fistule ou de brèche trachéale (Figure 2). La gastroscopie était en faveur d´une fundite érythémateuse, antrite érythémateuse blanchâtre, absence de brèche œsophagienne. Devant l´absence de perforation œsophagienne et la régression spontanée des symptômes, le diagnostic de syndrome de Boerhaave était écarté et le diagnostic de pneumomédiastin spontané au décours d´efforts de vomissements était retenu. L´évolution clinique était favorable après la mise en place d´un traitement symptomatique marqué par la disparition complète de l´emphysème cutanée, et l´évolution radiologique était marquée par la régression radiologique du pneumomédiastin (Figure 3).

## Discussion

La présentation clinique initiale du pneumomédiastin spontané peut évoquer celle d´une perforation œsophagienne ou trachéale [3]. Son évolution, est toujours bénigne, autorise une prise en charge minimale et la distingue des tableaux de fistule aérodigestive (notamment le syndrome de Boerhaave) dont la mortalité oscille aujourd´hui encore autour de 25% [4]. Cliniquement, le patient présente une douleur thoracique rétrosternale, d´installation brutale (70 à 90%). Elle est d´abord latéralisée du côté de la rupture alvéolaire, puis médiane, médiastinale. Les douleurs cervicales isolées sont beaucoup plus rares. La dyspnée est présente une fois sur trois, et l´emphysème sous-cutané, recherché attentivement, est palpé dans 60% des cas, mais n´apparaît qu´après 12 heures d´évolution [5]. Le motif de consultation de notre patiente correspondant à celui de la littérature.

Le mécanisme du pneumomediastin spontané, fait appel à un mécanisme appelé effet Macklin [6]. Il s´agit d´une rupture alvéolaire secondaire à une hyperpression intrabronchique brutale, due à des manœuvres de Valsalva [7]. L´air, après la rupture de la paroi alvéolaire, chemine le long des septas, rejoignant le médiastin par le hile et le ligament triangulaire créant ainsi le pneumomédiastin [8] puis il diffuse vers les espaces sous-cutanés cervicaux, péricardiques ou rétropéritonéaux à travers les voies de communication du médiastin. La diffusion de l´air à travers la plèvre viscérale peut expliquer la survenue concomitante d´un pneumothorax. Ces grands efforts à glotte fermée surviennent chez les asthmatiques, les parturientes et les grands sportifs Ils surviennent aussi au cours des efforts de toux intenses et des vomissements, et chez les consommateurs de drogue. Dans notre cas, le pneumo-médiastin spontané est survenu au cours des efforts de vomissements intenses.

La radiographie thoracique met en évidence un pneumo-médiastin ainsi que des signes d´emphysème sous-cutané; sa sensibilité varie entre 70 et 100% [9]. Le scanner thoracique est demandé soit de manière systématique [9] ou en cas de doute selon les équipes. Il confirme la présence d´air dans le médiastin et les tissus sous-cutanés si elle était passée inaperçue à la radiographie et met parfois en évidence un pneumothorax, un pneumopéritoine, un pneumopéricarde voire, dans certains cas exceptionnels, un pneumo rachis associé [10].

Les deux cas de pneumomédiastin spontané rapportés dans la littérature portent sur deux hommes, d´une vingtaine d´années, fumeurs, ayant comme antécédents un asthme [1]. L´élément déclenchant était plusieurs épisodes de vomissements. La conduite à tenir thérapeutique consiste à une surveillance rapprochée associant le repos, les antalgiques et oxygénothérapie en cas de dyspnée [9]. Un traitement de couverture antibiotique, généralement par céphalosporine de troisième génération, est recommandé [9]. La mise à jeun n´est pas systématique [9]. La durée d´hospitalisation varie entre 3 et 7 jours [10].

## Conclusion

Le pneumomédiastin est une entité rare et bénigne. Avant de retenir le diagnostic de ce dernier, il faut écarter la présence d´une atteinte digestive ou trachéale, dont la méconnaissance expose à des complications graves. La tomodensitométrie (TDM) thoracique permet de poser le diagnostic positif et aussi le suivi évolutif des patients, qui ne nécessitent aucune prise en charge thérapeutique.
